# Cyanidin-3-rutinoside acts as a natural inhibitor of intestinal lipid digestion and absorption

**DOI:** 10.1186/s12906-019-2664-8

**Published:** 2019-09-05

**Authors:** Thavaree Thilavech, Sirichai Adisakwattana

**Affiliations:** 1 Phytochemical and Functional Food Research Unit for Clinical Nutrition, Department of Nutrition and Dietetics, Faculty of Allied Health Sciences, Chulalongkorn University, Bangkok, 10330 Thailand; 2 Department of Food Chemistry, Faculty of Pharmacy, Mahidol University, Bangkok, 10400 Thailand

**Keywords:** Cyanidin-3-rutinoside, Anthocyanin, Cholesterol, Hyperlipidemia, Pancreatic lipase, Niemann-pick C1-like 1

## Abstract

**Background:**

Cyanidin-3-rutinoside (C3R), a naturally occurring anthocyanin, possesses anti-oxidant, anti-hyperglycemic, anti-glycation and cardioprotective properties. However, its mechanisms responsible for anti-hyperlipidemic activity have not been fully identified. The aim of the study was to investigate the lipid-lowering mechanisms of C3R through inhibition of lipid digestion and absorption in vitro.

**Methods:**

The inhibitory activity of C3R against pancreatic lipase and cholesterol esterase was evaluated using enzymatic fluorometric and enzymatic colorimetric assays, respectively. An enzyme kinetic study using Michaelis-Menten and the derived Lineweaver-Burk plot was performed to understand the possible types of inhibition. The formation of cholesterol micelles was determined using the cholesterol assay kit. The bile acid binding was measured using the colorimetric assay. The NBD cholesterol uptake in Caco-2 cells was determined using fluorometric assay. The mRNA expression of cholesterol transporter (Niemann-Pick C1-like 1) was determined by RT-PCR.

**Results:**

The results showed that C3R was a mixed-type competitive inhibitor of pancreatic lipase with the IC_50_ value of 59.4 ± 1.41 μM. Furthermore, C3R (0.125–1 mM) inhibited pancreatic cholesterol esterase about 5–18%. In addition, C3R inhibited the formation of cholesterol micelles and bound to primary and secondary bile acid. In Caco-2 cells, C3R (12.5–100 μM) exhibited a significant reduction in cholesterol uptake in both free cholesterol (17–41%) and mixed micelles (20–30%). Finally, C3R (100 μM) was able to suppress mRNA expression of NPC1L1 in Caco-2 cells after 24 h incubation.

**Conclusions:**

The present findings suggest that C3R acts as a lipid-lowering agent through inhibition of lipid digestion and absorption.

## Background

Hyperlipidemia is a group of metabolic disorders characterized by hypercholesterol and/or hypertriglyceride in blood circulation. The prevalence of hyperlipidemia has dramatically increased worldwide due to a sedentary lifestyle and consumption of high fat diets [1, 2]. Long-term hyperlipidemia is an important risk factor in contributing to the development of cardiovascular diseases (CVD). The risk of developing cardiovascular diseases in subjects with hyperlipidemia was twice as high as in subjects with normal lipid levels [3]. Currently, the inhibition of dietary fat digestion and absorption from the small intestine is an attractive target for the management of hyperlipidemia [4, 5]. For example, ezetimibe is a cholesterol absorption inhibitor that can be used as monotherapy or a combination therapy with a first-line drug such as a statin. Evidence has demonstrated that a combination of ezetimibe and a statin caused the lowering of low-density lipoprotein (LDL) cholesterol and improved cardiovascular outcomes in patients after acute coronary syndromes [6, 7]. However, the number of patients with elevated liver enzyme levels showed a significant increase with ezetimibe-statin combination therapy [8]. Therefore, current studies attempt to search for effective phytochemical compounds from dietary fruits, vegetables and herbal medicines as lipid-lowering agents [9, 10].

Phytochemical compounds in fruits and vegetables have been targeted to promote beneficial health effects, especially the prevention of pathophysiological conditions such as dyslipidemia, diabetes, hypertension and cancer [11, 12]. Anthocyanins are one of the largest groups of natural pigments responsible for red, purple and blue colors in fruits and vegetables. They have been proven to possess favorable antioxidant, anti-inflammatory and anti-diabetic properties under both in vitro and in vivo study [13]. Interestingly, a meta-analysis revealed that supplementation of anthocyanins reduced serum lipid profiles in dyslipidemia patients [14]. Cyanidin-3-rutinoside (C3R), a naturally occurring anthocyanin, is widely distributed in a high number of dietary sources, such as blackberry, mulberry and black raspberry [15, 16]. This compound has demonstrated promising benefits for reduction of postprandial glucose through inhibition of pancreatic α-amylase and intestinal α-glucosidase [17, 18]. Another study suggests C3R could inhibit the glucose uptake in Caco-2 cells [19]. In vitro data supported the underlying mechanism and indicated that C3R might modulate postprandial glycemia by inhibiting carbohydrate digestive enzymes and decreasing glucose transport in the small intestine. C3R also regulated glucose uptake and increased GLUT4 expression in 3T3-L1 adipocytes through activation of the PI3K/Akt pathways [20]. Our studies found that C3R inhibited monosaccharide- and methylglyoxal-induced protein glycation in bovine serum albumin [21, 22]. A previous study has shown the vascular relaxing activity of C3R and its protection against methylglyoxal-induced vascular dysfunction in rats [23]. However, available data on C3R mostly focused on its anti-diabetic and anti-glycation activity. Existing researches have never established the potential effectiveness of C3R in relation to lipid-lowering activity. Therefore, the objective of the study was to determine whether C3R could inhibit pancreatic lipase, cholesterol esterase, and the binding of bile acid as well as the reduction of cholesterol micellization. Since the blood cholesterol level is also influenced by absorption of cholesterol in the small intestine, the effect of C3R on cholesterol uptake in the enterocytes was also determined.

## Methods

### Chemicals and reagents

Porcine pancreatic lipase, 4-methylumbelliferyl oleate (4-MUO), oleic acid, phosphatidylcholine, glycodeoxycholic acid, taurodeoxycholic acid, taurocholic acid, porcine cholesterol esterase, orlistat, and *p*-nitrophenylbutylrate (*p*-NPB) were purchased from Sigma (St. Louis, MO, USA). Total bile acid colorimetric assay kit and cholesterol test kit were obtained from GenWay (San Diego, CA, USA) and HUMAN GmbH Co. (Wiesbaden, Germany), respectively. Cholestyramine resin was purchased from RUBIÓ (Barcelona, Spain). Ezetimibe was purchased from Merck, Sharp & Dohme (Kenilworth, NJ, USA). 22-(N-(7-nitrobenz-2-oxa-1,3-diazol-4-yl)amino)-23,24-bisnor-5-cholen-3-ol (NBD-cholesterol) and TRIzol reagent were obtained from Invitrogen (Eugene, OR, USA). Cyanidin-3-rutinoside chloride was synthesized from quercetin-3-rutinoside according to a previous study [24]. All other chemical reagents used in this study were analytical grade.

### Pancreatic lipase assay

Pancreatic lipase activity was determined according to a previous study with minor modifications [25]. The various concentrations of C3R were incubated with pancreatic lipase solution (50 U/mL) and 100 μM 4-MUO in Tris buffer, pH 6.9 (13 mM tris-HCl, 150 mM NaCl and 1.3 mM CaCl_2_) at 37 °C. After 30 min of incubation, the reactions were terminated by adding 100 μL of 0.1 M citrate buffer, pH 4.2. The amount of released 4-MUO was measured by using a spectrofluorometer at excitation wavelength 320 nm and emission wavelength 450 nm. Orlistat was used as a positive control for this study. The results were expressed as percentage of inhibition:

$$ \%\mathrm{Pancreatic}\ \mathrm{lipase}\ \mathrm{inhibition}=\left[\left(\left(\mathrm{Fc}-{\mathrm{F}}_{\mathrm{C}\mathrm{B}}\right)-\left({\mathrm{F}}_{\mathrm{S}}-{\mathrm{F}}_{\mathrm{S}\mathrm{B}}\right)/\left({\mathrm{F}}_{\mathrm{C}}-{\mathrm{F}}_{\mathrm{C}\mathrm{B}}\right)\right)\right]\times 100 $$

F_C_ was fluorescence intensity of control and F_CB_ was the fluorescence intensity of control blank. F_S_ and F_SB_ were the fluorescence intensity of C3R or orlistat and sample blank, respectively.

### Enzyme kinetic for pancreatic lipase inhibitory activity

To investigate the type of inhibition of pancreatic lipase activity, the enzymatic analysis was performed according to the above-mentioned reaction. Maintaining the quantity of pancreatic lipase at 50 U/mL, C3R (50–200 μM) was measured in various concentrations of 4-MUO (6.25–100 μM). The type of inhibition was calculated on the basis of Lineweaver-Burk reciprocally plotted data.

### Pancreatic cholesterol esterase assay

The pancreatic cholesterol esterase assay was slightly modified according to a previous method [25]. Briefly, various concentrations of C3R were incubated with a mixture containing 5.16 mM taurocholic acid, 0.2 mM *p*-NPB in 0.1 M sodium phosphate buffer, and pH 7.0 containing 100 mM NaCl. The reaction was initiated by adding porcine pancreatic cholesterol esterase (1 μg/mL) at 37 °C for 5 min. The absorbance was measured at 405 nm using a microplate reader. Gallic acid was used as a positive control for this study. The percentage of inhibition was calculated following the equation below:

$$ \%\mathrm{Inhibition}=\frac{\mathrm{Abs}\ \mathrm{Control}-\mathrm{Abs}\ \mathrm{Sample}}{\mathrm{Abs}\ \mathrm{Control}}\times 100 $$

### Cholesterol micellization

Artificial micelles were performed according to a previously published report [25]. The mixture (containing 2 mM cholesterol, 1 mM oleic acid and 2.4 mM phophatidlycholine) was dissolved in methanol and dried under nitrogen gas. Then the mixture was re-dissolved with 475 μL of 15 mM PBS containing 6.6 mM taurocholate salt, pH 7.4. The emulsion was sonicated twice for 30 min and incubated overnight at 37 °C. The various concentrations of C3R (25 μL) were added to the mixed micelle solution and incubated at 37 °C for 2 h. The mixture was then centrifuged at 12,000 rpm for 20 min. The supernatant was collected for the determination of cholesterol using the cholesterol test kit. The percentage of inhibition was calculated following the equation below:

$$ \%\mathrm{Inhibition}=\frac{\mathrm{Cholesterol}\ \mathrm{level}\ \mathrm{in}\ \mathrm{control}-\mathrm{Cholesterol}\ \mathrm{level}\ \mathrm{in}\ \mathrm{sample}}{\mathrm{Cholesterol}\ \mathrm{level}\ \mathrm{in}\ \mathrm{control}}\times 100 $$

### Bile acid binding assay

The bile acid binding assay was performed according to a previous study [25]. Glycodeoxycholic acid, taurodeoxycholic acid and taurocholic acid were used as bile acid in this experiment. Briefly, various concentrations of C3R were incubated with each bile acid (2 mM) containing 0.1 M PBS, pH 7 at 37 °C. After 90 min incubation, the mixtures were filtered through a 0.2 μm filter to separate the bound from the free bile acids. The bile acid concentration was measured using total bile acid colorimetric assay kit. The percentage of inhibition was calculated following the equation below:

$$ \%\mathrm{Inhibition}=\frac{\mathrm{Bile}\ \mathrm{acid}\ \mathrm{level}\ \mathrm{in}\ \mathrm{control}-\mathrm{Bile}\ \mathrm{acid}\ \mathrm{level}\ \mathrm{in}\ \mathrm{sample}}{\mathrm{Bile}\ \mathrm{acid}\ \mathrm{level}\ \mathrm{in}\ \mathrm{control}}\times 100 $$

### Cholesterol uptake in Caco-2 cells

Caco-2 cells were obtained from the American Type Culture Collection (ATCC®-HTB-37™). Cells were maintained in Dulbecco’s Modified Eagle’s Medium (DMEM) high glucose containing 10% fetal bovine serum, 1% penicillin/streptomycin, and 1% non-essential amino acids. Cells were cultured at 37 °C in a humidified incubator with 5% CO_2_ and seeded on a 24-well plate at a cell density of 25,000 cells/well for 7 days to allow them to differentiate. During this period, cells were fed with fresh medium every 2 days. After 7 days, cells were starved with serum-free DMEM low glucose containing 1% penicillin/streptomycin and 1% non-essential amino acids for 24 h. Then cells were washed twice with Hanks’ balanced salt solution (HBSS, pH 7.4, 140 mM NaCl, 5 mM KCl, 1.2 mM Na_2_HPO_4_, 2 mM CaCl_2_, 1.2 mM MgSO_4_, 20 mM HEPES, 0.2% bovine serum albumin) and incubated in HBSS at 37 °C before the experiments. After 1 h, cells were incubated at 37 °C for 1 h with free cholesterol (containing 0.5 mM taurocholate salt and 25 μM NBD-cholesterol) or mixed micelles (containing 0.5 mM taurocholate salt, 50 μM oleic acid, 20 μM phosphatidylcholine and 25 μM NBD-cholesterol) [26] as donor vehicles supplemented with C3R (12.5–100 μM). Ezetimibe (100 μM) was used as a positive control for this study. Cells were washed 5 times with cold HBSS and the fluorescence was measured at the excitation wavelength of 485 nm and emission wavelength of 535 nm. Cells were then disrupted in a lysis buffer (10 mM tris-HCl pH 7.4, 150 mM NaCl, 1% triton-x-100, 1 mM EDTA and 0.1% SDS) and lysed by the freeze-thaw method for 2 cycles. The lysates were centrifuged at 12,000 rpm at 4 °C for 10 min and the supernatants were collected for the determination of protein concentration using a BCA kit (Thermo Fisher Scientific, Waltham, MA, USA) with bovine serum albumin as standard. The total protein represented the total number of cells used for normalization. The results were expressed as the percentage of cholesterol uptake corresponding to the control values.

### Gene expression

Caco-2 cells were seeded on a 6-well plate at a cell density of 100,000 cells/well for 7 days to allow them to differentiate. During this period, cells were fed with fresh medium every 2 days. After 7 days, cells were treated with 100 μM C3R in serum-free medium, and then incubated at 37 °C for 2, 6 and 24 h. After incubation, RNA was extracted from cells using TRIzol reagent and converted to cDNA by ImProm-II™ Reverse Transcription System from Promega Corporation (Madison, WI, USA) according to the manufacturer’s direction. The specific primer sequences were obtained from previous studies as follows: Niemann-Pick C1-like 1 (NPC1L1, forward: 5′-TATGGTCGCCCGAAGCA-3′ and reverse: 5′-TGCGGTTGTTCTGGAAATACTG-3′) [27] and glyceroldehyde-3-phosphate dehydrogenase (GAPDH, forward: 5′-CATGAGAAGTATGACAACAGCCT-3′ and reverse: 5′-AGTCCTTCCACGATACCAAAGT-3′) [19]. RT-PCR was carried out in the CFX Connect™ Real-Time PCR Detection System (Bio-RAD Laboratories Inc., California, USA) using SsoFast™Evagreen Supermix SYRB green detection (Bio-RAD) according to the manufacturer’s instruction. All samples were carried out in triplicate. The values were normalized using GAPDH as an endogenous internal standard. The results were expressed as the level of relative quantity to control.

### Statistical analysis

Data are represented as means ± standard error of mean (SEM) for each group. The IC_50,_ *Ki* and *Ki’* values were calculated by using Sigma Plot 11.0. The multiple comparisons were analyzed by one-way ANOVA using Duncan post hoc. The effect of C3R on NPC1L1 gene expression at each incubation time was determined by Student’s t-test. These analyses were performed using SPSS Statistics 17.0 (SPSS Inc., Chicago, IL, USA). A *p* value of less than 0.05 was taken as the criterion of significance.

## Results

### Pancreatic lipase inhibition

**Fig. 1:**
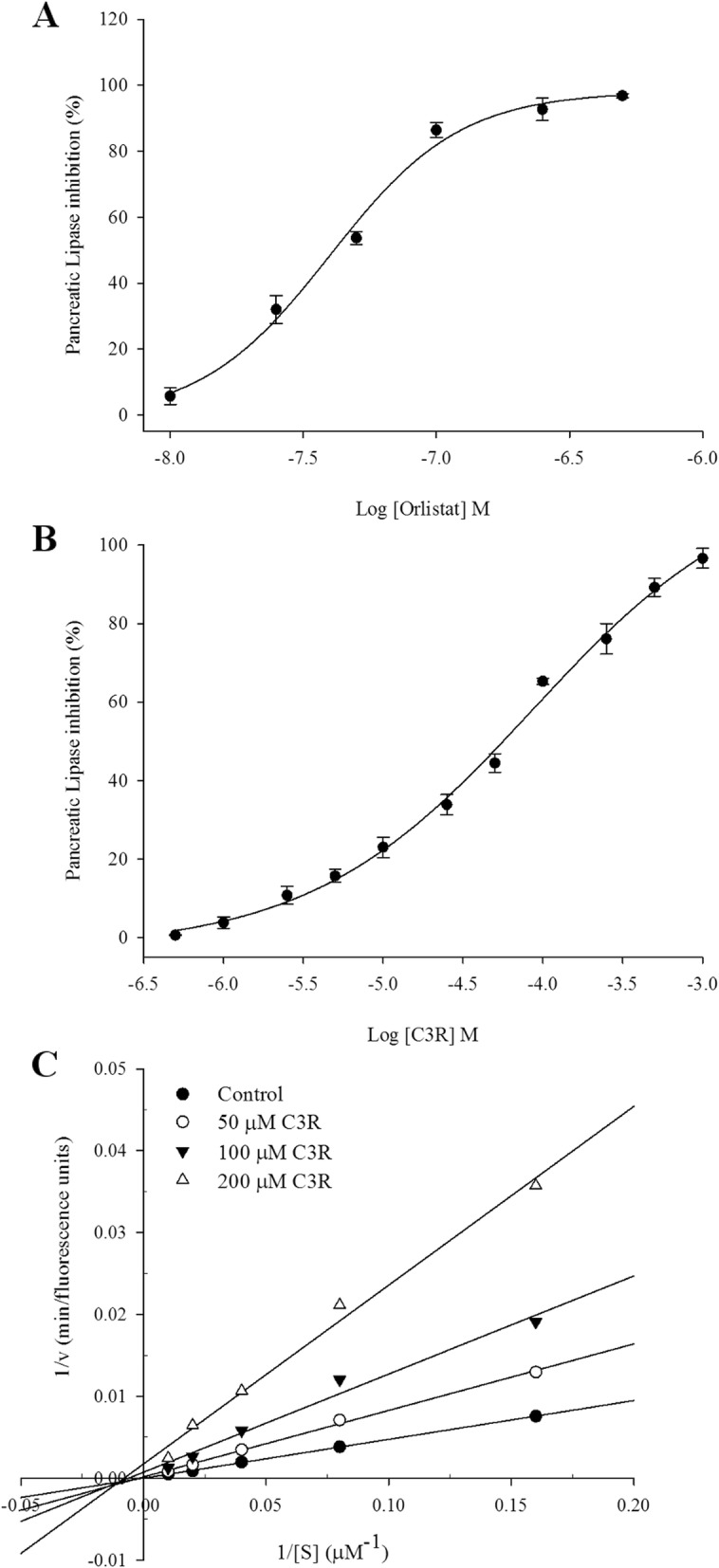
The concentration-response curves of orlistat (**a**) and cyanidin-3-rutinoside (**b**). **c** Lineweaver-Burk plot of cyanidin-3-rutinoside for pancreatic lipase inhibition. The results are presented as mean ± SEM (*n* = 3)

As shown in Fig. 1b, C3R strongly inhibited pancreatic lipase activity in a concentration-dependent manner (*p* < 0.05). The half-maximal inhibitory concentration (IC_50_) value of C3R against pancreatic lipase was 59.4 ± 1.41 μM, which was a lower potency than orlistat (IC_50_ = 31.7 ± 2.72 nM) (Fig. 1a).

To further explore the inhibitory characteristics of C3R, a kinetic inhibition study was performed using Lineweaver-Burk double reciprocal plots. As shown in Fig. 1b, a Lineweaver-Burk plot of C3R produced straight lines with different intersections for three concentrations of substrate. The lines for C3R had a point of intersection in the third quadrant, indicating that C3R activity was a mixed type competitive. The dissociation constant for C3R binding to free enzyme, *Ki*, was 39.0 ± 5.14 μM, while the dissociation constant for C3R binding with enzyme-substrate complex, *Ki’*, was 20.8 ± 9.92 μM.

### Cholesterol esterase inhibition

**Fig. 2:**
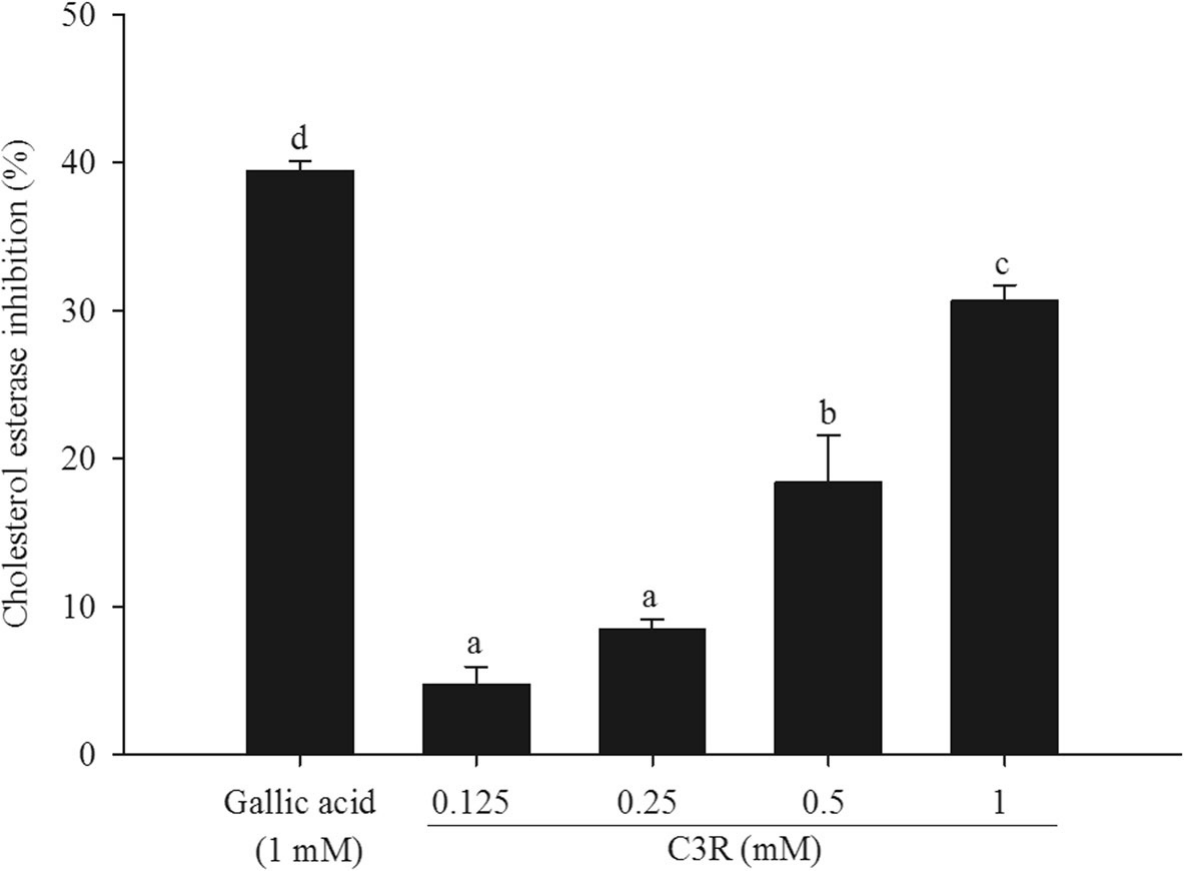
The percentage enzyme inhibition of cyanidin-3-rutinoside (C3R) on cholesterol esterase. The results are presented as mean ± SEM (*n* = 3). The groups that do not share a common letter are significantly different (*p* < 0.05)

As shown in Fig. 2, C3R significantly inhibited cholesterol esterase in a concentration-dependent manner. The percentage of the inhibition of C3R (0.125–1 mM) was 5–31%, whereas 1 mM gallic acid inhibited cholesterol esterase about 39%.

### Cholesterol micellization

**Fig. 3:**
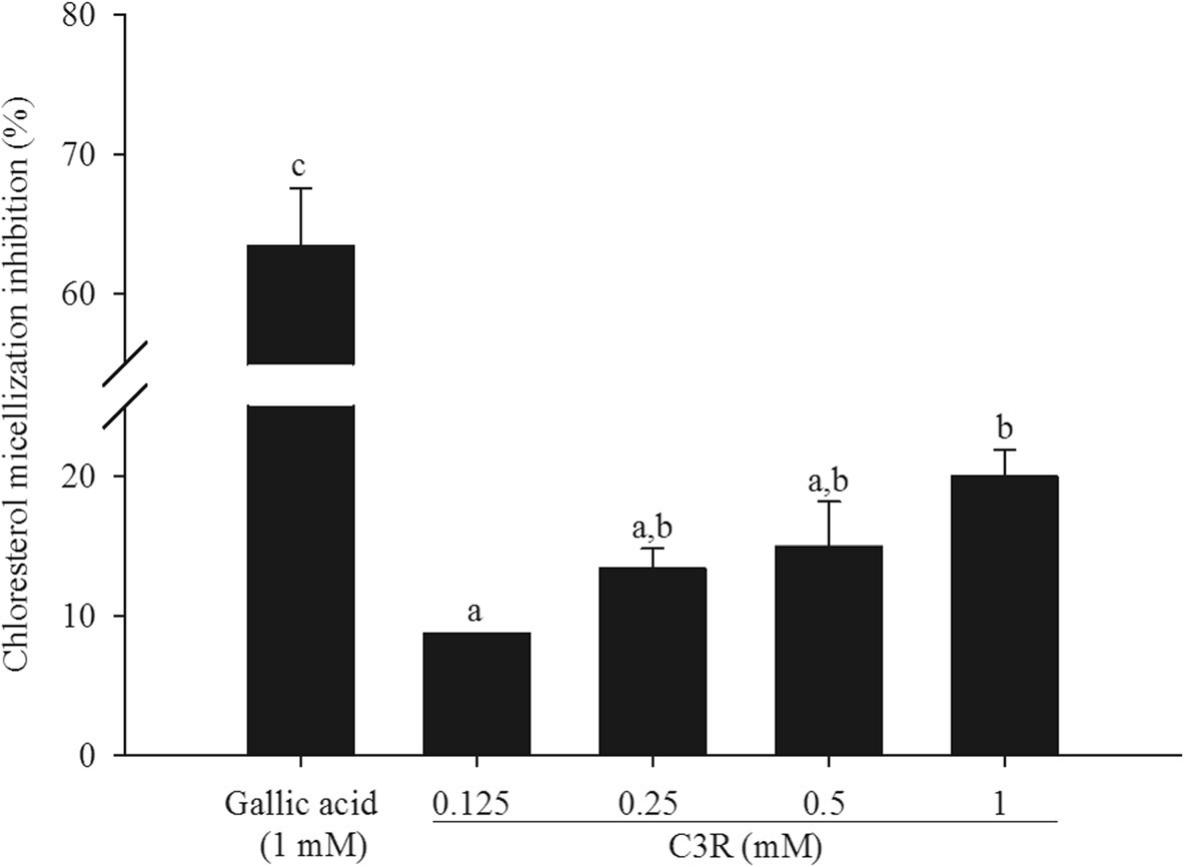
The percentage inhibition of cyanidin-3-rutinoside (C3R) on cholesterol micellization. The results are presented as mean ± SEM (*n* = 3). The groups that do not share a common letter are significantly different (*p* < 0.05)

Fig. 3 shows the percentage inhibition of C3R on cholesterol micellization. C3R (0.125–1 mM) markedly reduced the solubility of cholesterol in artificially prepared micelles by 9–20%, whereas gallic acid (1 mM) inhibited the formation of cholesterol micelles by about 64%.

### Bile acid binding

The percentage of taurocholic acid, taurodeoxycholic acid and glycodeoxycholic acid binding capacity of C3R is shown in Table 1. According to the results, C3R bound to bile acids in a concentration-dependent manner. When comparing the equal concentration of C3R (3 mM), the percentage of bile acid binding increased in the order of taurodeoxycholic acid (20%) < glycodeoxycholic acid (23%) < taurocholic acid (25%). However, C3R was less potent than cholestyramine at equal concentrations.

**Table 1:** The percentage binding of cyanidin-3-rutinoside (C3R) on bile acids

Experiments	Bile acid binding (%)								
	Taurocholic acid			Taurodeoxycholic acid			Glycodeoxycholic acid		
0.5 mM C3R	15.1	±	0.6^a^	13.0	±	0.3^a^	11.7	±	1.7^a^
1 mM C3R	18.2	±	0.9^a^	15.4	±	0.5^ab^	15.3	±	0.1^ab^
2 mM C3R	22.8	±	0.5^b^	16.4	±	0.9^b^	19.2	±	0.4^bc^
3 mM C3R	24.8	±	0.9^b^	19.5	±	0.4^c^	22.7	±	0.3^c^
3 mM Cholestyramine	37.1	±	0.1^c^	68.6	±	0.4^d^	60.2	±	1.0^d^

The results are expressed as mean ± SEM (n = 3). Groups without a common supercribed letter are significant difference (*p* < 0.05)

### Cholesterol uptake in Caco-2 cells

**Fig. 4:**
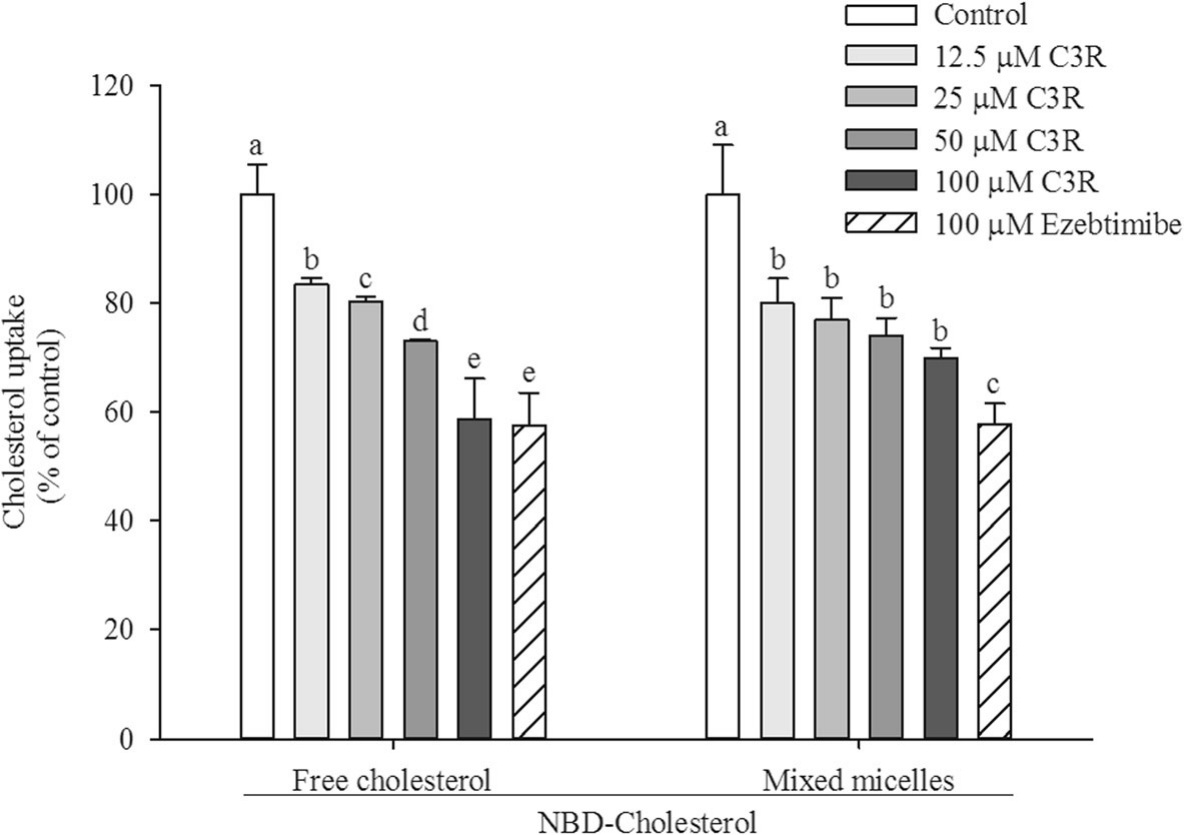
The effect of cyanidin-3-rutinoside (C3R) on cholesterol uptake in Caco-2 cells in form of free cholesterol and mixed micelles. The results are presented as mean ± SEM (n = 3). The groups that do not share a common letter are significantly different (*p* < 0.05)

The cholesterol absorption in Caco-2 cells was significantly suppressed by C3R in both forms of free cholesterol and mixed micelles (Fig. 4). The reduced cholesterol uptake was observed at the lowest concentration of C3R (12.5 μM). In free cholesterol, C3R inhibited cholesterol absorption in a concentration-dependent manner. When comparing at the same concentration (100 μM), C3R also decreased cholesterol absorption (41%) as well as ezetimibe (42%). However, C3R demonstrated a slight effect on suppression of mixed micelle cholesterol absorption (30%). It was found that ezetimibe had a higher potency than C3R on reduction of mixed micelle cholesterol uptake (42%).

### NPC1L1 gene expression

**Fig. 5:**
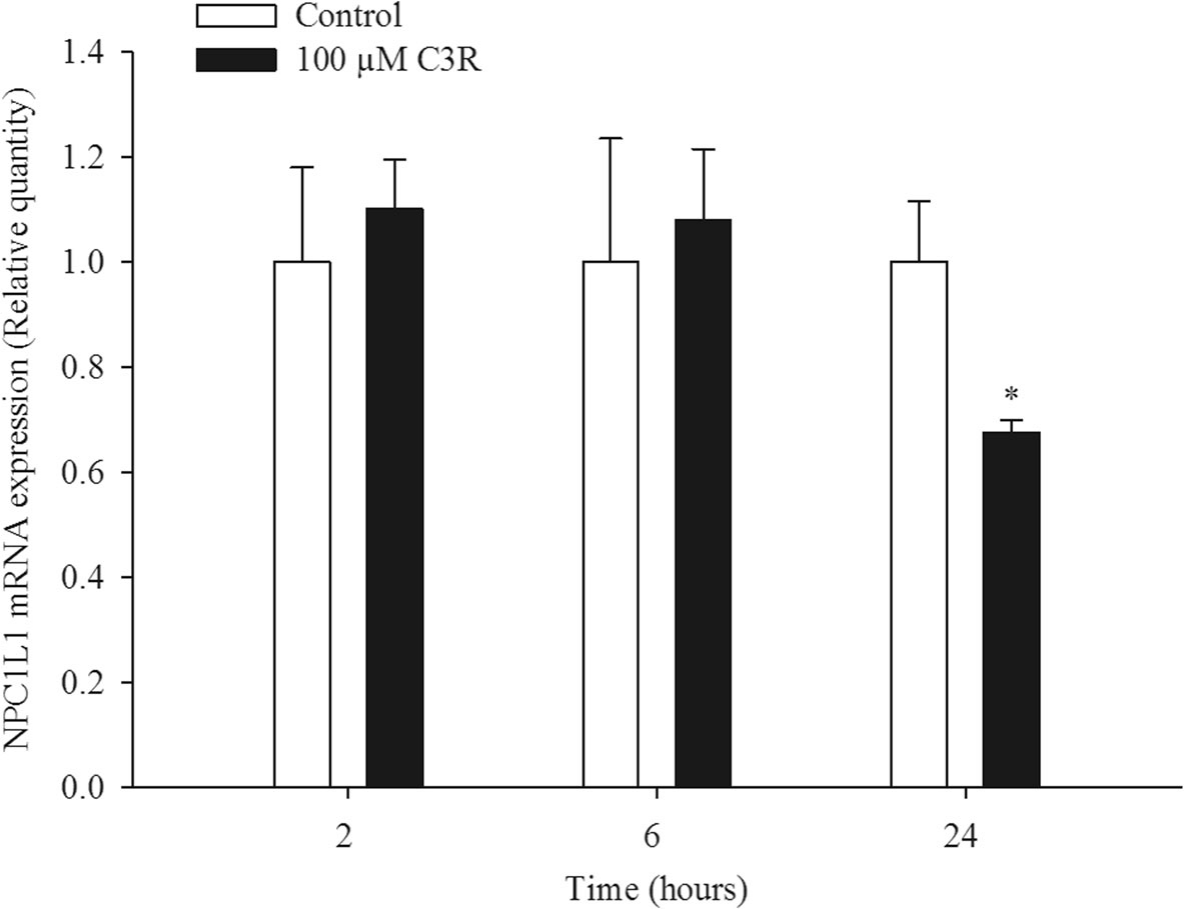
The effect of cyanidin-3-rutinoside (C3R; 100 μM) on Niemann-Pick C1-Like 1 (NPC1L1) mRNA expression in Caco-2 cells after 2, 6, and 24 h incubation. The results are presented as mean ± SEM (n = 3). *p < 0.05 compared to the control

The effects of C3R on NPC1L1 gene expression are shown in Fig. 5. There are no significant differences of NPC1L1 gene expression between the control and C3R after 2 and 6 h incubation. However, C3R caused a 7-fold suppression in NPC1L1 gene expression after 24 h incubation.

## Discussion

Clinical evidence revealed that supplementation of anthocyanin reduced total cholesterol, triglyceride and the level of low-density lipoprotein cholesterol (LDL-C) and increased the level of high-density lipoprotein cholesterol (HDL-C) in patients with dyslipidemia [28, 29]. The previous study reported that consumption of purified mulberry anthocyanins containing cyanidin-3-rutinoside (C3R) effectively decreased serum lipid levels in high-fat fed mice [30]. However, the specific mechanisms of action by which C3R decreases serum lipid level are still unknown. We present the first report on lipid-lowering mechanisms of C3R, including inhibition of lipid digestive enzymes and absorptive processes.

Inhibition of fat digestive enzymes results in delaying the process of hydrolyzing dietary fats. Pancreatic lipase is the primary digestive enzyme that converts triglyceride substrates to monoglycerides and free fatty acids, which may form micelles that serve as necessary intermediates for absorption into enterocytes [4]. The inhibition of pancreatic lipase activity is the most widely studied mechanism for the identification of potential anti-obesity agents [4]. The results from the pancreatic lipase inhibitory activity of C3R are in agreement with previous reports suggesting that pure anthocyanins, cyanidin-3-glucoside (C3G) and peonidin-3-glucoside, and anthocyanins extracted from dietary sources demonstrated inhibitory action against pancreatic lipase [31, 32]. Since understanding the mechanism of enzyme inhibition has become the basis of development of pharmaceutical agents, the study of the structure-enzyme activity relationship provides helpful information for drug design [33]. According to the results, C3R displayed a mixed-type competitive inhibitor against pancreatic lipase. The binding mode of C3R was assumed to be one inhibitor that can bind either to the active site of a free enzyme or to the enzyme-substrate complex. When *K*_*i*_ and *K*_*i*_*’* dissociation constants were compared, it was found that the *K*_*i*_ value of C3R was 1.9 times higher than the *K*_*i*_*’* value, suggesting that binding of C3R to a free form of enzyme was stronger than the binding of C3R to enzyme-substrate complex. The results indicated that the pancreatic lipase inhibitory activity of C3R was non-competitive predominant over competitive. However, the type of inhibition of C3R differs from earlier reports that cyanidin and its glycosides, including C3G and cyanidin-3,5-diglycoside, were identified as a competitive inhibitor [31, 34]. It has been reported that cyanidin and C3G had three potential binding sites for porcine lipase-colipase complex within and near the active site, and cyanidin bound more effectively than C3G within (− 9.8 vs. -7.0 kcal/mol) and near (− 8.8 vs. -8.0 kcal/mol) the active site [35]. Therefore, the different type of enzyme inhibition might be due to their glycosides at the 3-*O*-position of cyanidin, which may affect the binding site and binding affinity of the anthocyanins to the enzyme [36]. Therefore, further study is needed to evaluate the interaction between pancreatic lipase and C3R containing rutinose sugar at the 3-*O*-position using the molecular docking study.

Pancreatic cholesterol esterase plays an important role in the hydrolyzing of dietary cholesterol ester (10–15% of total cholesterol in food) into non-esterified cholesterol which can be incorporated into mixed micelles and absorbed by enterocytes [37]. It has been reported that the inhibition of pancreatic cholesterol esterase caused a reduction in cholesterol absorption in hamsters fed a high cholesterol diet [38]. The current findings firstly revealed that C3R inhibited pancreatic cholesterol esterase activity, which is consistent with previous reports describing the potency of plant and fruit extracts against cholesterol esterase [25, 39]. It has been stated that the structure of flavonoids is similar to that of cholesterol ester, which could irreversibly bind to the active site of the enzyme, resulting in inhibition of enzyme activity [40]. According to the basic structural feature of flavonoid compounds, C3R may act in the same manner.

Generally, the principle steps in absorption of dietary cholesterol are emulsification of dietary fat, micellization of cholesterol, and absorption of mixed micelles in the proximal jejunum [41]. One of the cholesterol-lowering mechanisms of flavonoids (tea catechins) is the interruption of cholesterol incorporation into micelles [42]. The present study indicated that micellization of cholesterol was inhibited by C3R. In support of this, the recent study revealed that cyanidin-3-glucoside (C3G) added to micellar solution precipitated with cholesterol and formed an insoluble complex [31]. Therefore, it could be hypothesized that C3R may prevent the formation of micelles due to having the same core chemical structure as C3G with a different type of sugar at the β-glycosidic linkage.

In addition to cholesterol, bile acids are also essential components in the formation of mixed micelles [41]. C3R exhibited the binding ability of bile acids, including taurocholic acid, taurodeoxycholic acid and glycodeoxycholic acid. C3R showed the strongest binding capacity to taurocholic acid, a primary bile acid directly synthetized by the liver, suggesting that C3R may disrupt the endogenous bile acid pool, causing the stimulation of bile acid synthesis from cholesterol and leading to the reduction of blood cholesterol [43]. Moreover, the binding of bile acids causes the formation of an insoluble complex and increases fecal bile excretion as well as disrupts the formation of micelles [43]. Taken together, these findings support the notion that C3R may act as the bile acid sequestrant, resulting in increased cholesterol metabolism and decreased cholesterol absorption into blood circulation.

Intestinal Caco-2 cells are a widely used human intestinal in vitro model that obtain results that usually correlate with human figures [44]. Since intestinal cholesterol absorption affects the levels of cholesterol in blood circulation, the effect of C3R on cholesterol uptake was determined in Caco-2 cells [44]. Our results demonstrated that C3R inhibited cholesterol uptake in both free cholesterol (containing only bile salt taurocholate) and mixed micelles (mimicking the mixed micelles found in dietary conditions). These results indicated that the action of C3R was independent of cholesterol micelle formation, and the reduction of cholesterol uptake was not due to a toxic effect of C3R on cell viability (data not shown). The results are consistent with previous reports that anthocyanins in black rice extract are a potent agent to reduce cholesterol uptake in enterocytes [31]. Interestingly, C3R showed sustained inhibitory effects similar to ezetimibe. This agent has a distinct mechanism as a selective cholesterol uptake inhibitor through binding of NPC1L1 at the brush border of the small intestine [45]. Our results found that anthocyanin C3R markedly decreased mRNA expression of NPC1L1 in Caco-2 cells. Nevertheless, the short-term mechanism by which C3R inhibits cholesterol uptake does not appear to relate to the suppression of NPC1L1 mRNA expression in Caco-2 cells. This conclusion is drawn from the fact that a significant decrease in NPC1L1 mRNA expression was observed only at 24 h incubation with C3R, whereas there was no significant alteration of NPC1L1 mRNA expression at 2 h and 6 h. We suggest that C3R may interact with NPC1L1 protein to inhibit the cholesterol uptake after a short exposure period [45]. In addition, the suppression of NPC1L1 mRNA expression by C3R appears to be the long-term mechanism for reducing cholesterol uptake into the enterocytes.

## Conclusions

**Fig. 6:**
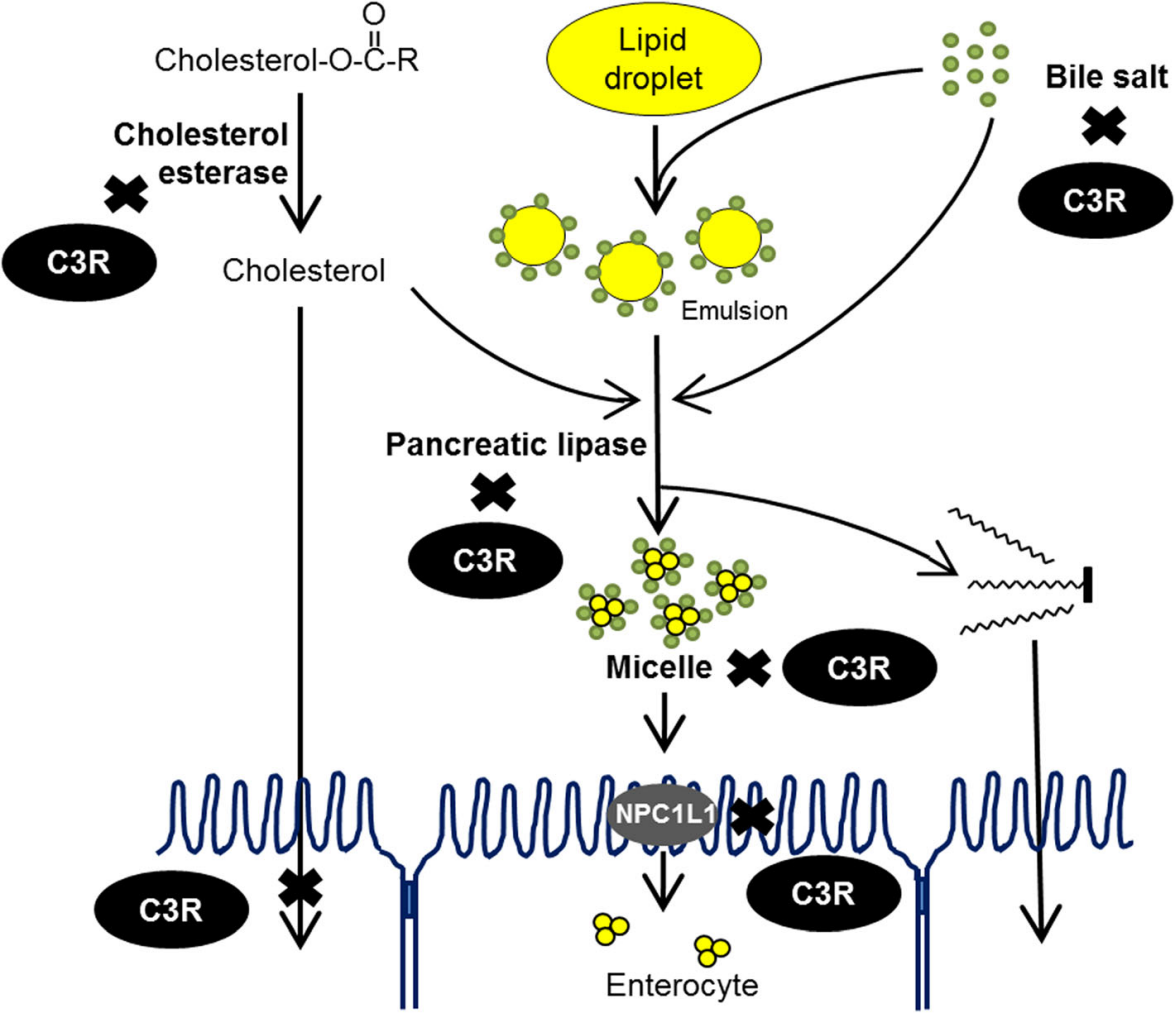
A schematic illustrations of lipid-lowering actions of cyanidin-3-rutinoside (C3R)

C3R inhibited pancreatic lipase and cholesterol esterase activity as well as cholesterol micellization. It also bound various types of bile acid (Fig. 6). In addition, C3R also inhibited cholesterol uptake in the enterocytes in both free cholesterol and mixed micelles. The inhibition may involve in the down-regulation of NPC1L1 mRNA expression. The findings suggest that C3R may be an effective compound, with promising anti-hyperlipidemic properties.

## Data Availability

The datasets used and/or analysed during the current study available from the corresponding author Dr. Sirichai Adisakwattana (sirichai.a@chula.ac.th) on reasonable request.

## Abbreviations

4-MUO: 4-methylumbelliferyl oleate
C3G: Cyanidin-3-glucoside
C3R: Cyanidin-3-rutinoside
GAPDH: Glyceroldehyde-3-phosphate dehydrogenase
IC_50_: The half maximal inhibitory concentration
NBD-cholesterol: 22-(N-(7-nitrobenz-2-oxa-1,3-diazol-4-yl)amino)-23,24-bisnor-5-cholen-3-ol
NPC1L1: Niemann-Pick C1-Like 1
*p*-NPB: *p*-nitrophenylbutylrate
RT-PCR: Real time-polymerase chain reaction
